# Two SARS-CoV-2 Genome Sequences of Isolates from Rural U.S. Patients Harboring the D614G Mutation, Obtained Using Nanopore Sequencing

**DOI:** 10.1128/MRA.01109-20

**Published:** 2020-12-17

**Authors:** Piroon Jenjaroenpun, Visanu Wanchai, Kikumi D. Ono-Moore, Jennifer Laudadio, Laura P. James, Sean H. Adams, Fred Prior, Intawat Nookaew, David W. Ussery, Thidathip Wongsurawat

**Affiliations:** a Department of Biomedical Informatics, College of Medicine, University of Arkansas for Medical Sciences, Little Rock, Arkansas, USA; b Division of Bioinformatics and Data Management for Research, Department of Research and Development, Faculty of Medicine, Siriraj Hospital, Mahidol University, Bangkok, Thailand; c Arkansas Children's Nutrition Center, Little Rock, Arkansas, USA; d Department of Pathology, University of Arkansas for Medical Sciences, Little Rock, Arkansas, USA; e Department of Pediatrics, College of Medicine, University of Arkansas for Medical Sciences, Little Rock, Arkansas, USA; DOE Joint Genome Institute

## Abstract

Two coding-complete sequences of severe acute respiratory syndrome coronavirus 2 (SARS-CoV-2) were obtained from samples from two patients in Arkansas, in the southeastern corner of the United States. The viral genome was obtained using the ARTIC Network protocol and Oxford Nanopore Technologies sequencing.

## ANNOUNCEMENT

As the novel coronavirus disease 2019 (COVID-19) outbreak continues to worsen around the world, the daily death toll in the United States is currently averaging more than 1,000 deaths per day. Rapid sharing of genome sequences in conjunction with other epidemiological data can facilitate early decision-making in an attempt to control the local transmission of severe acute respiratory syndrome coronavirus 2 (SARS-CoV-2), an RNA virus that belongs to the genus *Betacoronavirus*, in the family *Coronaviridae*. In this work, we used Oxford Nanopore Technologies (ONT) MinION sequencing technology, which provided a consensus viral genome from SARS-CoV-2-positive samples within 1 day. Importantly, the device can be easily used in environments with very limited resources, such as in rural areas without access to traditional laboratory facilities.

A set of two residual, deidentified nasopharyngeal samples (USA/AR-UAMS001/2020 and USA/AR-UAMS002/2020) that tested positive for SARS-CoV-2 by quantitative reverse transcription-PCR (qRT-PCR) were obtained from patients at the University of Arkansas for Medical Sciences (UAMS) hospital. Total RNA was extracted by the QIAamp viral RNA minikit (Qiagen, USA) according to the manufacturer’s instructions. Samples were reverse transcribed as described in the PCR tiling of COVID-19 virus protocol (vPTC_9096_v109_revF_06Feb2020) published by the ARTIC Network (https://www.protocols.io/view/ncov-2019-sequencing-protocol-v3-locost-bh42j8ye). The PCR amplification process was slightly modified from the ARTIC Network protocol by changing the annealing and extension temperature from 65°C to 63°C. The libraries were prepared using a ligation-based sequencing kit (SQK-LSK109 kit; ONT), loaded onto a MinION flow cell (ONT), and sequenced with the MinION Mk1B device (ONT). Base calling of the resulting FAST5 files was performed in real time using Guppy (v3.4.5) (1) on a MinIT device (ONT) using the high accuracy mode. The RAMPART software (v1.0.5) from the ARTIC Network (https://github.com/artic-network/rampart) was used to monitor sequencing in real time. The minimum coverage we used for each region on the genome was 300×. For quality control and filtering of reads (fragments of 400 to 700 bp), the guppyplex script of the ARTIC Network bioinformatics protocol (https://artic.network/ncov-2019/ncov2019-bioinformatics-sop.html) was used, followed by a reference assembly using the MinION script with madeka polishing against the sequence of the Wuhan-Hu-1 isolate (GenBank accession number MN908947.3). The quality metrics for the reference-based assemblies are shown in Table 1. Based on the ARTIC Network primer sets, the sequencing did not cover 54 bases from the 5′ end and 67 bases from the 3′ end of the virus reference genome. All samples were obtained with the approval of the institutional review board (IRB) at UAMS (IRB approval number 260840) and were processed by the Center for Molecular Diagnostics at UAMS.

**TABLE 1:** Assembly metrics and accession numbers for two SARS-CoV-2 genomes

Sample	Total sequenced bases (Gb)	Total no. of sequenced reads	GenBank accession no.	Genome size (bp)	Minimum coverage (×)	GC content (%)
USA/AR-UAMS001/2020	1.9	4,921,525	MT766907.1	29,782	373	38
USA/AR-UAMS002/2020	2.2	5,429,747	MT766908.1	29,782	613	38

The data sets of 4,114 SARS-CoV-2 genomes deposited in GISAID (sampled between December 2019 and July 2020) were used for phylogenetic analysis. The phylogenetic analysis was performed following the standard protocol for analysis of SARS-CoV-2 genomes provided by Nextstrain (http://nextstrain.org/ncov) (2). We used MAFFT v7.471 for alignment and implemented the rapid phylodynamic alignment pipeline provided by Augur (2). A maximum-likelihood phylogenetic tree was reconstructed using IQ-TREE (v1.5.5) with the general time-reversible (GTR) model (3).

**FIG 1:**
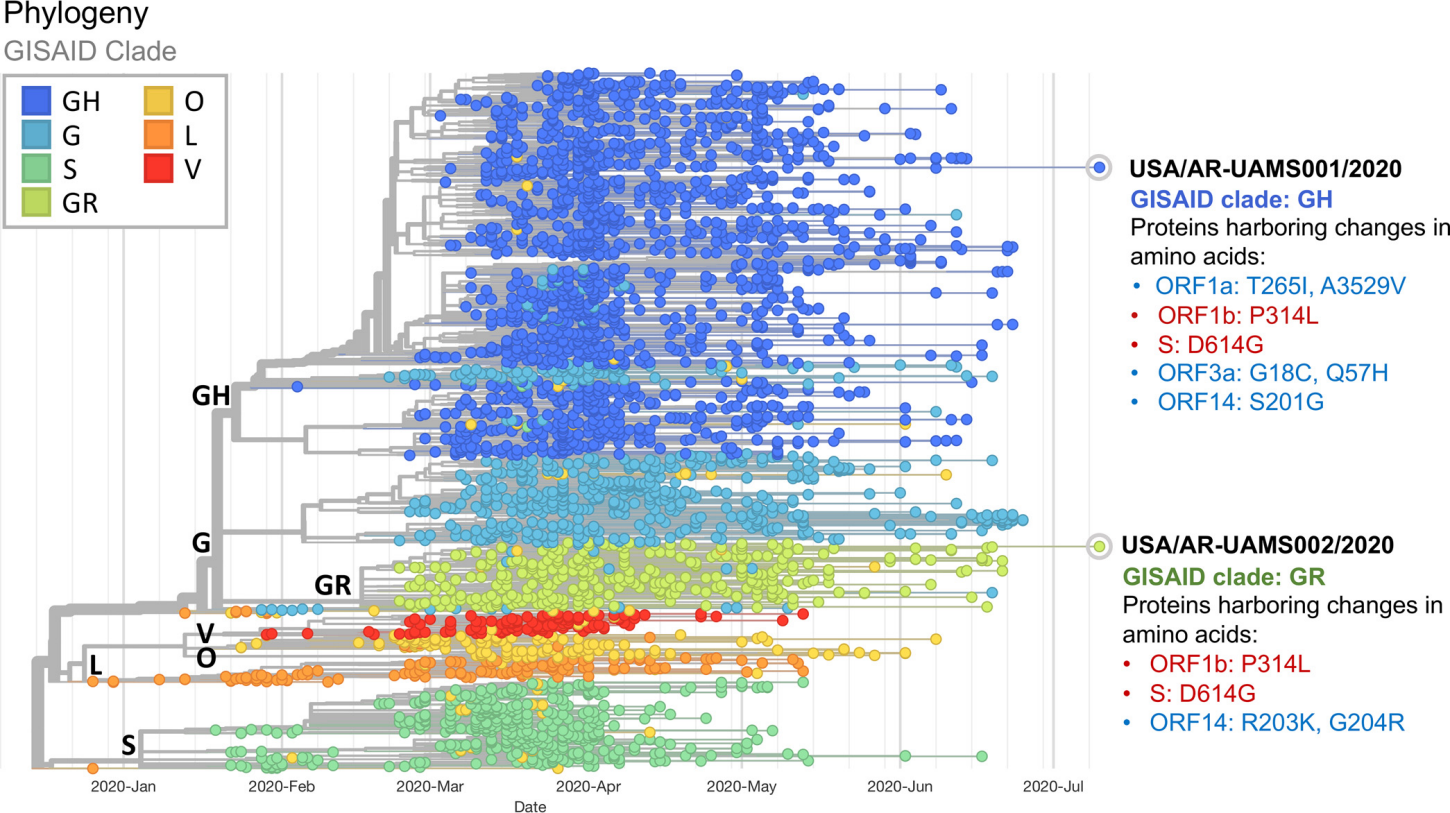
Phylogenetic analysis of SARS-CoV-2 representative genome sequences, including two UAMS genomes collected in Arkansas. Available genomes were retrieved from GISAID (https://www.gisaid.org) on 7 July 2020. The color (clade) of the dots was classified according to the mutation marks from the GISAID database nomenclature. We discarded sequences with low quality, i.e., ambiguous bases. The figure was created using Nextstrain. Seven mutations were found in USA/AR-UAMS001/2020, and four mutations were found in USA/AR-UAMS002/2020. Unique mutations between the two strains are shown in blue letters, and common mutations for each strain are shown in red letters.

Figure 1 shows the genetic relationship between the USA/AR-UAMS001/2020 and USA/AR-UAMS002/2020 isolates and other strains in the GISAID database. Both isolates were grouped in clade G (S protein D614G mutation) but in different subclusters, i.e., USA/AR-UAMS001/2020 was grouped in SARS-CoV-2 clade GH (open reading frame 3a [ORF3a] Q57H mutation), while USA/AR-UAMS002/2020 was grouped in clade GR (ORF14 G204R mutation). Genomes containing D614G mutations of spike protein are now enriched among recent SARS-CoV-2 isolates (4). A recent study (July 2020) by Mercatelli and Giorgi shows that clade GH is much more prevalent than other types in North America and clade GR is currently the most common representative of the SARS-CoV-2 population worldwide (5). The origin of the two UAMS strains, derived from Arkansas residents and belonging to distinct clades, remains unknown. Regardless, the results highlight that, despite the higher or lower relative prevalence of GH versus GR clade genomes in viruses sampled within and outside North America, each clade is present within the different populations. There were five unique mutations found in the first isolate (USA/AR-UAMS001/2020); two were found in ORF1a (T265I and A3529V), two in ORF3a (G18C and Q57H), and one in ORF14 (S201G). In contrast, there were only two unique mutations found in the second isolate (USA/AR-UAMS002/2020); both were found in ORF14 (R203K and G204R).

### Data availability.

The coding-complete sequences of the two isolates were deposited in GenBank (GenBank accession number MT766907 and SRA accession number SRR12277392 for USA/AR-UAMS001/2020 and GenBank accession number MT766908 and SRA accession number SRR12277391 for USA/AR-UAMS002/2020) and in the Cancer Imaging Archive (TCIA) (6). The GISAID accession numbers are EPI_ISL_492181 for USA/AR-UAMS001/2020 and EPI_ISL_492182 for USA/AR-UAMS002/2020. The sequences can be downloaded from GISAID (www.gisaid.org).
